# The Medical Witness

**Published:** 1903-07-25

**Authors:** 


### The Medical Witness.

The smart saying about the three degrees of liars, in which the superlative place is given to "expert witnesses," touches only a small part of medical evidence. The theory on which expert evidence is employed presupposes that it is possible to form an opinion from certain data provided without knowing anything about the case in point from first hand. Assuming that the facts are such and such, they may mean this or that for the issue that is being tried; and, if the parties in the cause have long purses, they can usually get one or more "experts" to favour their respective contentions. Thus there arises what is called "a conflict of medical opinion." But the fault is really with the Law, because it assumes that the matters of fact may be ascertained by one order of medical men and reasoned upon by another. Nebuchadnezzar relates a complicated dream, and Daniel interprets it for him. In the diagnosis or prognosis of difficult cases everyone knows that the solution of the problem depends really upon a full and minute knowledge of the facts at first hand. That is the meaning of Cullen's aphorism, "that there are more false facts than false theories." One observer may notice something that another has missed; and that one fact, even a small one, may change the whole aspect of the case. On the other hand, to take somebody's data, perhaps scrappy notes or oral depositions before a magistrate, and to ask two or more experts to say how they bear upon the issue at trial, is to raise an inevitable "conflict of medical testimony."

So long as the law upholds the characteristic legal fiction that an expert opinion can be given upon a paper medical case, so long will medical testimony have a bad name. In the business of coroners' courts, which may or may not come to criminal trial or to an action for damages, there should always be medical agreement. In Germany it is so. The post-mortem is made by two competent medical men, who agree as to the notes (the so-called "protocol") before they leave the room and agree in their judgment. The accused or suspected party is never "represented at the post-mortem"; the facts are assumed to be safe in the hands of two men, in the presence of a legal official. Where with us an accused person is "represented" his representative (who is probably paid much better than the working pathologist) may do nothing to help out the truth; he lies low, thinking all the while how he can wrest the facts to suit his client's case. This is an invidious position for a medical man to be placed in, but it is in accordance with English maxims of justice to accused persons; and here, again, the profession of Medicine is made the scapegoat of the Law. If the same case were made the subject of a clinical lecture, or of a paper before a medical society, we should have the physician or surgeon in his scientific frame of mind; if the case becomes the subject of an action for damages, or of a criminal trial, we have him in his forensic frame of mind. Yet he is not an advocate: he is a witness, who is sworn to behave as if he were a scientific person.

The medical witness who wishes, as he mostly does, to tell the court exactly what he knows and believes is often sorely tried by the tortuous ways of counsel. Hypothetical questions are framed, which assume what is not in the case, but might have been. This is a common line of defence with solicitors in the first stages of a trial. You say that the accused was drunk, but would not locomotor ataxia account for his unsteady gait? Would not the rupture of an aneurism account for the blood which you have spoken to? Another way to bother the truthful witness is to misstate the facts in an ingenious way, and elicit a new opinion, which is correct for the facts as misstated, but is at variance with what the witness had said before. There is no escape from this species of boredom. What he wishes to answer all the time, but is not allowed to, is that the questions do not arise in the actual circumstances of the case. He must rack his brains to imagine all kinds of things, which he knows to be irrelevant, if not positively untrue, or even impossible. One great safeguard of his common sense lies in avoiding the technicalities which medical men use with each other, and conveying his information in such a form as the jury will understand.

